# Diagnostic value of the γ-glutamyltransferase and alanine transaminase ratio, alpha-fetoprotein, and protein induced by vitamin K absence or antagonist II in hepatitis B virus-related hepatocellular carcinoma

**DOI:** 10.1038/s41598-020-70241-5

**Published:** 2020-08-11

**Authors:** Guangrong Wang, Xiaolan Lu, Qin Du, Guoyuan Zhang, Dongsheng Wang, Qiang Wang, Xiaolan Guo

**Affiliations:** 1 Department of Laboratory Medicine, Affiliated Hospital of North Sichuan Medical College, Nanchong, Sichuan People’s Republic of China; 2 Faculty of Laboratory Medicine, Center for Translational Medicine, North Sichuan Medical College, Nanchong, Sichuan People’s Republic of China; 3 Department of Laboratory Medicine, North Sichuan Medical College, Nanchong, Sichuan People’s Republic of China

**Keywords:** Biomarkers, Oncology

## Abstract

Hepatocellular carcinoma is a common type of malignancy with a poor prognosis. Identification and utilisation of markers for monitoring and diagnosis are urgently needed. Alpha-fetoprotein (AFP) and Protein Induced by Vitamin K Absence or Antagonist-II (PIVKA-II) have been proved to be efficient biomarkers for hepatitis B virus (HBV)-related hepatocellular carcinoma (HCC). The combination of the two markers could improve the detection rate. However, these indicators cannot meet the need of clinical diagnosis.It is necessary to discover novel serological markers and more cost-effective, appropriate combination of these markers for the diagnosis and surveillance of HBV-related HCC. Accordingly, in this study, we aimed to evaluate the diagnostic value of γ-glutamyltransferase (γ-GT) to alanine amino transferase (ALT) ratio alone or in combination with AFP and PIVKA-II for HBV-related HCC. 234 patients with HBV-related HCC and 396 patients with chronic hepatitis B (CHB) were enrolled in this study and approved by the institutional review board. Our results showed levels of AFP and PIVKA-II, and γ-GT/ALT ratio in cases with early-stage HCC, HCC, HCC plus HBV DNA positivity, and HCC plus HBV DNA negativity were higher than those in the corresponding CHB control group. Additionally, the levels of serum AFP and PIVKA-II, and the γ-GT/ALT ratio were positively correlated with tumour sizes in patients with HBV-related HCC. The areas under the ROC curves (AUROCs) of the γ-GT/ALT ratio in patients with early-stage HCC, HCC, HCC plus HBV DNA positivity, and HCC plus HBV DNA negativity were 0.795, 0.846,0.855, and 0.837, respectively; AUROCs of combination of the γ-GT/ALT ratio and PIVKA-II were 0.858, 0.928, 0.948, and 0.902, respectively; AUROCs of combination of the γ-GT/ALT ratio and AFP were 0.822, 0.886, 0.896, and 0.873, respectively;AUROCs of combination of the γ-GT/ALT ratio and PIVKA-II with AFP were 0.857, 0.928, 0.946, and 0.907, respectively, and AUROCs of combination of PIVKA-II and AFP were 0.804, 0.904, 0.942, and 0.863, respectively. In conclusion, the γ-GT/ALT ratio was a useful biomarker for the diagnosis of HBV-related HCC and that the combination of AFP and PIVKA-II with the γ-GT/ALT ratio could improve the diagnostic value of these biomarkers for HBV-related HCC. Moreover, the ratio of γ-GT/ALT may be a useful index in monitoring patients for progression of HBV-related HCC.

## Introduction

Hepatocellular carcinoma (HCC) is the sixth most common type of malignant tumour and the third leading cause of cancer-related death^1^. The occurrence of HCC can be caused by many factors, including hepatitis B virus (HBV)/hepatitis C virus (HCV) infection and alcohol consumption^2^. Among these factors, HBV infection is most closely related to the occurrence of HCC, accounting for 75–80% of virus-related HCC^3^. Attribute to HBV infection, the incidence of HCC is higher in China than the average incidence worldwide^4–8^, about 330,000 people die of HCC every year^9–11^. Therefore, there is no doubt that the health and economic burden of HBV-related HCC is particularly challenging and serious in China. Morever, about 50% HCC cases diagnosed are at advanced stage when curative treatments are very limited, and 5-year survival rate is considerably low^12^. Thus, screening, early detecting, and diagnosing of HCC among patients with CHB is urgently needed.

Currently, Alpha-fetoprotein (AFP) and protein induced by vitamin K absence or antagonist II (PIVKA-II) are widely used biomarkers in HCC diagnosis^13–16^. These markers have been written into the guidelines for HCC diagnosis published by the National Society of Hepatology of different countries^17–19^. However, owing to increased AFP levels in patients with hepatitis and cirrhosis, not all patients with HCC show high levels of AFP, and approximately 40% of patients with HCC have negative AFP^20^. However, the sensitivity and specificity of AFP in the diagnosis of HCC are not satisfactory, making AFP unsuitable for clinical applications^21^. PIVKA-II, also known as demethoxy prothrombin, is a type of prothrombin that does not use vitamin K for hepatocyte synthesis, resulting in deficiencies in synthesis and absence of coagulation function. When HCC occurs, the absence of vitamin K-dependent carboxylase in malignant hepatocytes leads to insufficient carboxylation of prothrombin precursor, leading to accumulation of PIVKA-II^22^. As a tumour marker for the diagnosis of HCC, PIVKA-II has a higher diagnostic value than AFP^14,23^, particularly for AFP-negative HCC or early-stage HCC^24–26^^.^. Some studies have shown that PIVKA-II has a sensitivity of more than 90% for HCC diagnosis, although its sensitivity for early-stage HBV-related HCC detection is less than 70%^23,27–29^.

γ-Glutamyl transpeptidase (γ-GT) is a membrane-binding enzyme that is essential for glutathione synthesis and is considered a biomarker of liver cell damage^30^. γ-GT in serum is mainly derived from the liver, and the level of γ-GT is abnormally elevated in various hepatobiliary diseases. Many studies have shown that intrahepatic obstruction leads to cholestasis, which induces the liver to produce large amounts of γ-GT in patients with HCC, and tumour cells can produce γ-GT themselves; thus, the level of γ-GT in serum is abnormally elevated in patients with HCC. However, in other liver diseases, such as viral hepatitis, alcoholic hepatitis, and cirrhosis, the level of γ-GT in serum is also abnormally elevated^31–34^. Therefore, the specificity of γ-GT in the diagnosis of HCC is only approximately 30%^35^. Based on these analyses, γ-GT cannot be used as an effective index to diagnose HCC, but could be used as an effective indicator for evaluation of patient liver function. Alanine aminotransferase (ALT) is an enzyme that participates in the metabolism of human proteins. ALT is widely expressed in various tissues and organs in humans, with the highest expression observed in the mitochondria of hepatocytes. When 1% of hepatocytes are destroyed, ALT is released into the blood, thereby increasing the activity of ALT in the blood. Therefore, this protein is often used as a biomarker to evaluate damage to hepatocytes. In acute and chronic viral hepatitis, alcoholic hepatitis, and cirrhosis, the level of ALT is elevated to varying degrees, whereas in patients with HCC, ALT is not elevated or is even decreased^13,36^.

Accordingly, in this study, we evaluated whether the ratio of γ-GT to ALT (γ-GT/ALT ratio) could be used to differentiate HBV-related HCC from CHB by increasing the gap between HCC and CHB. We also examined whether this ratio could be applied to the diagnosis and evaluation of HBV-related HCC and explored whether the combination of the γ-GT/ALT ratio and PIVKA-II and AFP, as known biomarkers for the diagnosis of HCC, could improve the diagnostic value of PIVKA-II and AFP for HBV-related HCC.

## Materials and methods

### Study setting and patients

In total, 630 patients with CHB, including 234 patients with HBV-related HCC and 396 patients with CHB with other liver diseases, were retrospectively enrolled at the Affiliated Hospital of Northern Sichuan Medical College from January 2017 to November 2018. HBV-related HCC patients were divided into the following categories: Early-stage HCC patients, HCC patients, HCC patients plus HBV DNA positivity (HBV DNA+), and HCC patients plus HBV DNA negative (HBV DNA−). Among the 234 patients with HCC, 94 had early-stage HCC, 110 had HCC with HBV DNA+, and 124 had HCC with HBV DNA−. The diagnosis of HCC was made in accordance with the standards of the guidelines for the diagnosis and treatment of primary HCC issued by the Chinese Society of Clinical Oncology (2018.V1). Early-stage HCC was defined as the presence of only a single tumour in the liver that was less than or equal to 5.0 cm, with no vascular invasion and extrahepatic metastasis^14^. Of the 396 patients with CHB, 190 patients had cirrhosis, 285 patients were HBV DNA+, and 111 patients were HBV DNA−. Diagnoses of CHB infection and cirrhosis were carried out in accordance with the revised guidelines for the prevention and treatment of CHB infection from the Chinese Society of Hepatology^37^.

### Measurement of γ-GT, ALT, AFP, PIVKA-II, and HBV DNA

Serum levels of γ-GT and ALT were determined using a biochemical rate-assay (AU5800; Beckman Coulter, Inc., USA). Serum levels of AFP were measured using an electrochemiluminescence immunoassay (Cobas E602; Roche, Inc., Germany). Serum levels of PIVKA-II were determined using a chemiluminescent microparticle immunoassay (Architect i1000; Abbott Laboratories, USA). Serum levels of HBV DNA were determined by real-time polymerase chain reaction (LightCycler 480II; Roche, Inc.). The results were interpreted as follows: HBV DNA ≥ 500 IU/mL, positive (HBV DNA+); and HBV DNA ˂ 500 IU/mL, negative (HBV DNA−)^16^.

### Data processing for the combined evaluation of γ-GT/ALT, PIVKA-II, and AFP

The receiver operating characteristic (ROC) curves were used to determine the diagnostic cut-off values of the γ-GT/ALT ratio, AFP, and PIVKA-II for HBV-related HCC. The fold changes in the serum levels of the γ-GT/ALT ratio, AFP, and PIVKA-II relative to the corresponding diagnostic cut-off values were expressed using the M_cut-off_. We evaluated the diagnostic values of the combined detection of biomarkers in HBV-related HCC by analysing the sums of the M_cut-off_ of the corresponding biomarkers.

### Statistical analysis

Data are expressed as the median (interquartile range) or number (%). Two groups were compared using Mann–Whitney U tests. Pearson’s chi-square tests were used for comparisons of sex. Pearson correlation analysis was used for two-factor correlation analysis. ROC curves were used to determine the diagnostic cut-off values, area under the ROCs (AUROCs), diagnostic sensitivities, and specificities. Statistical analyses were performed using SPSS, version 19.0 (SPSS Inc., USA) and Medcalc, version 12.3 (MedCalc Software bvba, Ostend, Belgium). Results with *P* values of less than 0.05 by two-tailed t tests were considered significant.

### Ethical approval and informed consent

The study protocol was approved by the Ethics Committee of the Affiliated Hospital of North Sichuan Medical College, Nanchong, China. All study participants provided informed consent and all methods were performed in accordance with the relevant guidelines and regulations of the committee.

## Results

### Comparison of clinical characteristics between the HCC and CHB control groups

The clinical characteristics of 234 patients with HCC and 396 patients with CHB (the control group) are shown in Table 1. The data showed that patients in both groups were predominately male and that the median age of patients with HCC was 54 (47–63) years, which was higher than that in the CHB control group (median 48 years [range 39–59 years]; *P* < 0.001). The serum levels of PIVKA-II, AFP, and γ-GT, and serum γ-GT/ALT ratio in patients with HCC were higher than those in CHB control patients (*P* < 0.001). In contrast, serum levels of ALT were lower in patients with HCC than in CHB controls (*P* < 0.001).

**Table 1:** Characteristics of the study population (n = 630).

	CHB (n = 396)	HCC (n = 234)	*P* value
Age (years)	48 (39–59)	54 (47–63)	< 0.001
Sex (male:female)	311:85	201:33	0.031
PIVKA-II (mAU/mL)	22.32 (15.96–36.84)	4,191.74 (294.12–24,580.91)	< 0.001
AFP (ng/mL)	30.45 (5.32–117.43)	576.30 (22.53–10,543.48)	< 0.001
γ-GT(U/L)	92.60 (46.03–162.85)	153.70 (74.18–313.30)	< 0.001
ALT(U/L)	154.60 (44.25–538.25)	47.00 (29.75–84.00)	< 0.001
γ-GT/ALT	0.57 (0.23–1.25)	3.22 (1.44–5.93)	< 0.001
HBV DNA+, n (%)	285 (71.97%)	110 (47.01%)	NA
Early-stage HCC, n (%)	NA	94 (40.17%)	NA

Data are expressed as medians (interquartile ranges) or numbers (%).

*CHB* chronic hepatitis B, *HCC* hepatocellular carcinoma, *PIVKA-II* protein induced by vitamin K absence or antagonist II, *AFP* alpha-fetoprotein, *γ-GT* γ-glutamyltransferase, *ALT* alanine transaminase, *NA* not applicable.

### Serum PIVKA-II and AFP levels and the serum γ-GT/ALT ratio in patients with CHB, early-stage HCC, and advanced HCC

**Figure 1:**
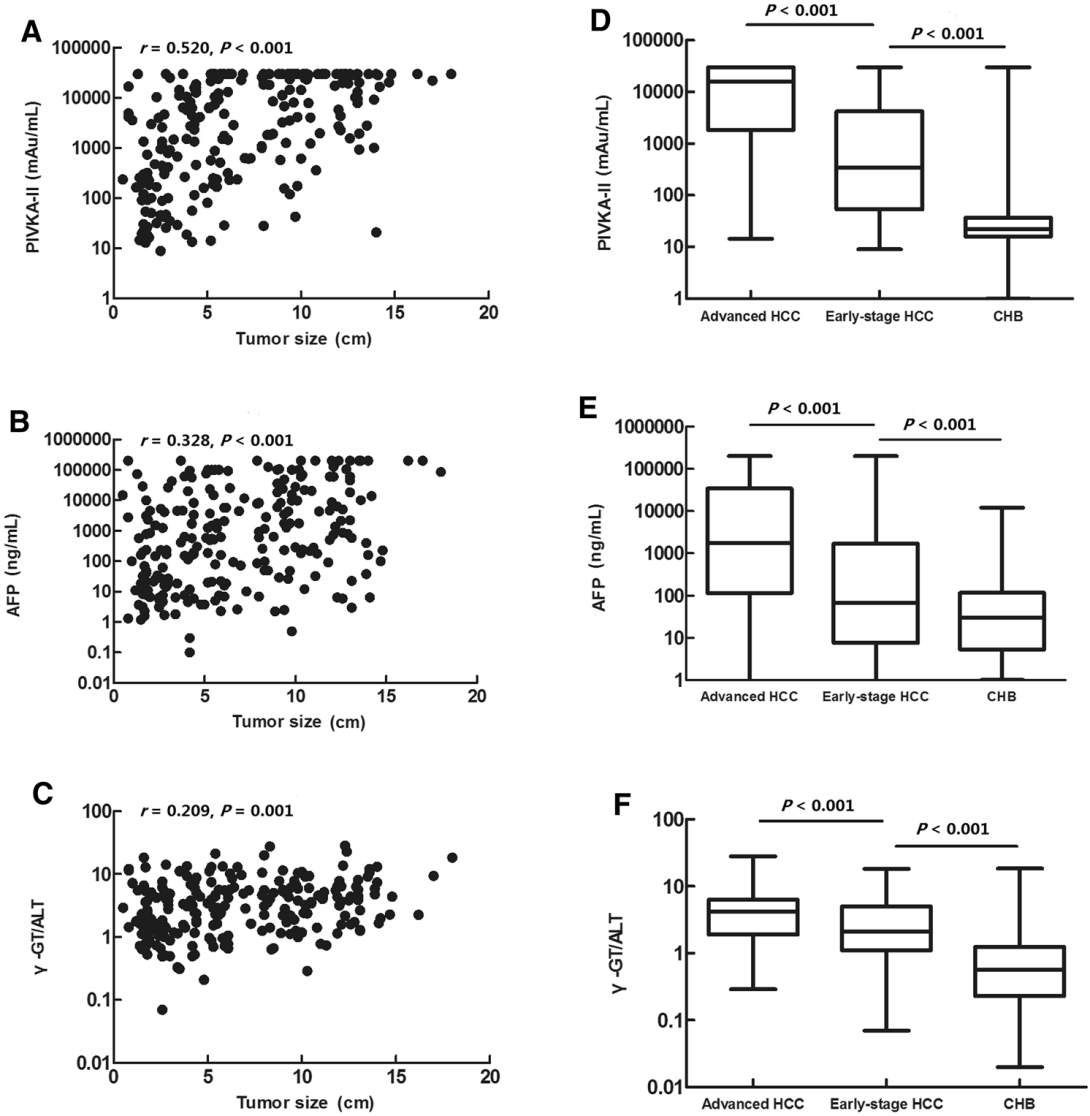
(**A**–**C**) Correlations of PIVKA-II, AFP, and the γ-GT/ALT ratio with tumour size. (**D**–**F**) Comparison of serum levels of PIVKA-II and AFP and the serum γ-GT/ALT ratio in patients with advanced HCC, early-stage HCC, and CHB.

Serum PIVKA-II and AFP levels and the serum γ-GT/ALT ratio were positively correlated with tumour size in patients with HCC (r = 0.520, *P* < 0.001; r = 0.328, *P* < 0.001; r = 0.209, *P* = 0.001, respectively; Fig. 1A–C). The serum levels of PIVKA-II and AFP and the serum γ-GT/ALT ratio in patients with early-stage HCC were significantly lower than those in patients with advanced HCC (*P* < 0.001). Both groups displayed significantly higher serum levels of PIVKA-II and AFP and higher serum γ-GT/ALT ratios compared with those in controls with CHB (*P* < 0.001; Fig. 1D–F, Table 2).

**Table 2:** Serum levels of PIVKA-II and AFP and the serum γ-GT/ALT ratio in patients with CHB, early-stage HCC, and advanced HCC.

	CHB (n = 396)	Early-stage HCC (n = 94)	Advanced HCC (n = 140)	*P* value
PIVKA-II (mAU/mL)	22.32 (15.96–36.84)	342.19 (53.56–4,208.13)^a^	15,701.03 (1,843.90–30,000.00)^ab^	0.000
AFP (ng/mL)	30.45 (5.32–117.43)	66.20 (7.63–1,718.80)^a^	1,767.60 (115.83–33,996.28)^ab^	0.000
γ-GT/ALT	0.57 (0.23–1.25)	2.13 (1.11–5.03)^a^	4.15 (1.91–6.36)^ab^	0.000

Data are expressed as medians (interquartile ranges).

*CHB* chronic hepatitis B, *HCC* hepatocellular carcinoma, *PIVKA-II* protein induced by vitamin K absence or antagonist II, *AFP* alpha-fetoprotein, *γ-GT/ALT* the ratio of γ-glutamyltransferase to alanine aminotransferase.

^a^Versus CHB.

^b^Versus early-stage HCC.

### Diagnostic value of γ-GT/ALT ratio, AFP and PIVKA-II in early-stage HCC patients

**Figure 2:**
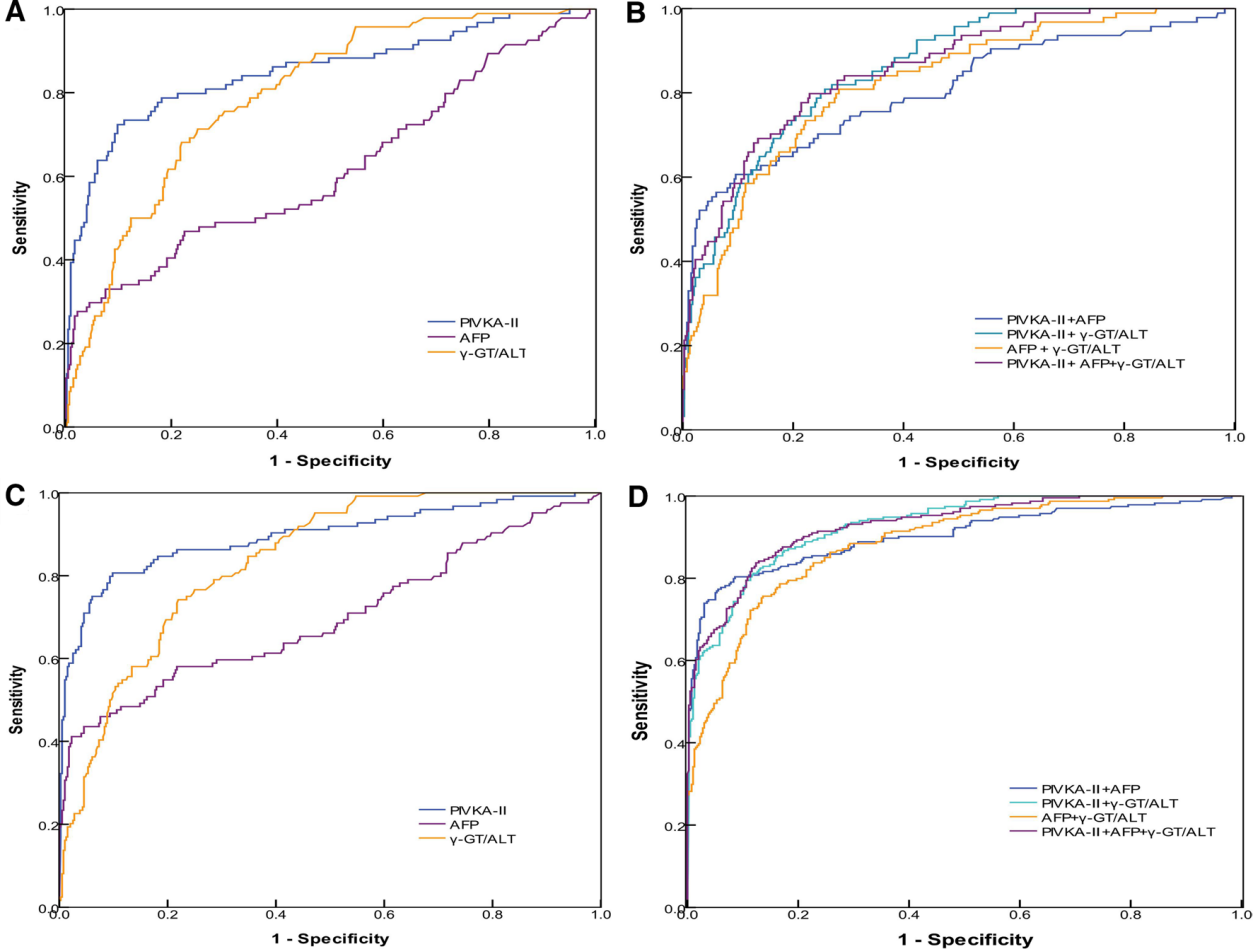
(**A**, **B**) ROC curves of PIVKA-II, AFP, the γ-GT/ALT ratio, and combinations of various biomarkers in patients with early-stage HCC; patients with CHB served as controls. (**C**, **D**) ROC curves of PIVKA-II, AFP, the γ-GT/ALT ratio, and combinations of various biomarkers in patients with HCC; patients with CHB served as controls.

When the cut-off values of the γ-GT/ALT ratio, AFP and PIVKA-II levels were set as 1.245, 556.90 ng/mL, and 87.63 mAu/mL, respectively, for the diagnosis of early-stage HCC, the AUROC of the γ-GT/ALT ratio was 0.795, which was higher than that of AFP (0.617; *P* < 0.001; Fig. 2A).

When combined with AFP in early-stage HCC, the AUROC of PIVKA-II decreased (0.804 versus 0.851, respectively). However, the difference was not significant between combined diagnosis and single diagnosis (*P* = 0.228). When combined with the γ-GT/ALT ratio for the diagnosis of early-stage HCC, the AUROC of PIVKA-II increased (0.858 versus 0.851, respectively), and when combined with the γ-GT/ALT ratio for early-stage HCC diagnosis, the AUROC of AFP increased (0.822 versus 0.617, respectively; *P* < 0.001). When PIVKA-II was combined with the γ-GT/ALT ratio and AFP for diagnosis of early-stage HCC, there was almost no difference between AUROCS for combined and single diagnosis (0.857 versus 0.851, respectively; *P* = 0.858; Fig. 2A,B).

#### Diagnostic value of PIVKA-II, AFP, and the γ-GT/ALT ratio in patients with HCC

When the cut-off values of the γ-GT/ALT ratio, AFP and PIVKA-II levels were set as 1.395, 499.80 ng/mL, and 87.63 mAu/mL, respectively, the AUROC of PIVKA-II for diagnosing HCC was significantly higher than that of AFP and γ-GT/ALT (*P* < 0.001, *P* < 0.001, respectively), whereas the AUROC of the γ-GT/ALT ratio for diagnosing HCC was significantly higher than that of AFP (*P* < 0.001; Fig. 2C). Compared with the results in patients with early-stage HCC, the diagnostic value of AFP and PIVKA-II increased in patients with advanced HCC (*P* = 0.002, *P* = 0.010); however, there were no significant differences in the diagnostic value of the γ-GT/ALT ratio between early-stage HCC and advanced HCC (*P* = 0.072).

When combined with AFP in HCC, the AUROC of PIVKA-II decreased (0.904 versus 0.925, respectively), and there were no significant differences in AUROCs between combined diagnosis and single diagnosis (*P* = 0.255). When combined with the γ-GT/ALT ratio for the diagnosis of HCC, the AUROC of PIVKA-II increased (0.928 versus 0.925, respectively). When combined with the γ-GT/ALT ratio for HCC diagnosis, the AUROC of the γ-GT/ALT ratio and AFP increased (0.886 versus 0.846 and 0.886 versus 0.745, respectively; *P* = 0.044, *P* < 0.001, respectively). When PIVKA-II was combined with the γ-GT/ALT ratio and AFP for diagnosis of HCC, there were no significant differences between combined and single diagnosis (0.928 versus 0.925, respectively; *P* = 0.848; Fig. 2C,D). The diagnostic performance values of PIVKA-II, AFP, and the γ-GT/ALT ratio for early-stage HCC and HCC are shown in Table 3.

**Table 3:** Performance value of PIVKA-II, AFP, and the γ-GT/ALT ratio in patients with early-stage HCC and HCC.

	Early-stage HCC						HCC					
	Cut-off	AUROC (95% CI)	SEN (%)	SPE (%)	PPV (%)	NPV (%)	Cut-off	AUROC (95% CI)	SEN (%)	SPE (%)	PPV (%)	NPV (%)
PIVKA-II (mAu/mL)	87.63	0.851 (0.801–0.901)	72.30	90.20	63.55	93.21	87.63	0.925 (0.901–0.950)	86.80	90.20	83.88	92.01
AFP (ng/mL)	556.90	0.617 (0.547–0.687)	33.00	92.40	50.82	85.31	499.80	0.745 (0.702–0.787)	52.10	91.40	78.21	76.37
γ-GT/ALT	1.245	0.795 (0.748–0.841)	71.30	75.00	40.36	91.67	1.395	0.846 (0.816–0.876)	77.40	78.00	67.54	85.36
PIVKA-II & AFP	NA	0.804 (0.748–0.860)	60.60	90.40	60.00	90.63	NA	0.904 (0.876–0.931)	80.30	91.40	84.68	88.73
PIVKA-II& γ-GT/ALT	NA	0.858 (0.820–0.895)	80.90	74.20	42.70	94.23	NA	0.928 (0.908–0.947)	85.50	83.80	75.76	90.71
AFP & γ-GT/ALT	NA	0.822 (0.775–0.868)	80.90	71.70	40.43	94.04	NA	0.886 (0.859–0.912)	78.60	83.30	73.60	86.84
PIVKA-II & AFP & γ-GT/ALT	NA	0.857 (0.816–0.898)	79.80	77.00	45.18	94.14	NA	0.928 (0.908–0.949)	83.80	87.60	80.00	90.13

*HCC* hepatocellular carcinoma, * SEN* sensitivity, * SPE* specificity, * PPV* positive predictive value, * NPV* negative predictive value, * PIVKA-II* protein induced by vitamin K absence or antagonist II, * AFP* alpha-fetoprotein, * γ-GT/ALT* ratio of γ-glutamyltransferase to alanine transaminase, * NA* not applicable.

### Serum levels of PIVKA-II and AFP and the serum γ-GT/ALT ratio in patients with HCC according to HBV DNA status

**Figure 3:**
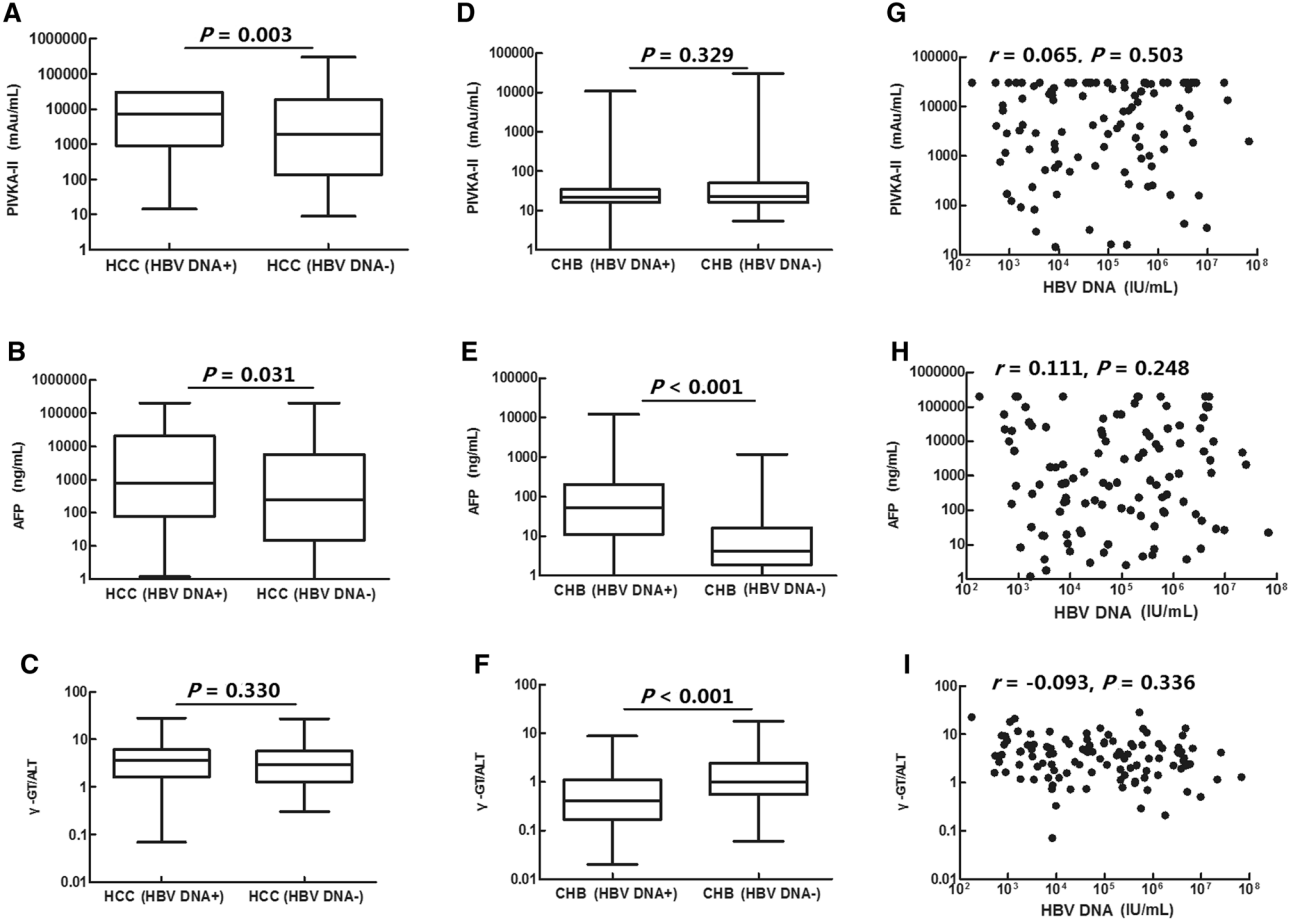
Correlation of serum levels of PIVKA-II and AFP and the serum γ-GT/ALT ratio with HBV DNA. (**A**–**C**) Serum levels of PIVKA-II and AFP and the serum γ-GT/ALT ratio were compared in patients with HCC who were HBV DNA+ and HBV DNA−. (**D**–**F**) Serum levels of PIVKA-II and AFP and the serum γ-GT/ALT ratio were compared in patients with CHB who were HBV DNA+ and HBV DNA−. (**G**–**I**) Correlations of serum levels of PIVKA-II and AFP and the serum γ-GT/ALT ratio with serum levels of HBV DNA in patients with HCC who were HBV DNA+.

Serum levels of AFP and PIVKA-II in patients with HCC were higher in those positive for HBV DNA than in those negative for HBV DNA (*P* = 0.031 and *P* = 0.003, respectively). There were no significant differences in γ-GT/ALT ratios between the two groups (*P* = 0.330; Fig. 3A–C, Table 4). Serum levels of AFP in patients with CHB positive for HBV DNA were significantly higher than those in patients with CHB negative for HBV DNA (*P* < 0.001; Fig. 3E). In contrast, the γ-GT/ALT ratio in patients with CHB was significantly lower in patients positive for HBV DNA than in patients negative for HBV DNA (*P* < 0.001; Fig. 3F); there were no significant differences in PIVKA-II levels between the two groups (*P* = 0.329; Fig. 3D). Serum levels of PIVKA-II and AFP and the serum γ-GT/ALT ratio were not correlated with serum levels of HBV DNA in patients with HCC who were positive for HBV DNA (*P* = 0.503, *P* = 0.248, and *P* = 0.336, respectively; Fig. 3G–I).

**Table 4:** Serum levels of PIVKA-II and AFP and the serum γ-GT/ALT ratio in patients with HCC according to HBV DNA status.

	HBV DNA+/HCC (n = 110)	HBV DNA−/HCC (n = 124)	*P* value
PIVKA-II (mAU/mL)	7,363.10 (922.13–30,000.00)	1,883.99 (132.38–18,618.54)	0.003
AFP (ng/mL)	796.75 (76.28–20,710.95)	247.20 (14.80–5,783.58)	0.031
γ-GT/ALT	3.63 (1.64–6.05)	2.92 (1.28–5.70)	0.330

Data are expressed as medians (interquartile ranges).

*HCC* hepatocellular carcinoma, *PIVKA-II* protein induced by vitamin K absence or antagonist II, *AFP* alpha-fetoprotein, *γ-GT/ALT* the ratio of γ-glutamyltransferase to alanine aminotransferase.

### Diagnostic value of PIVKA-II, AFP, and the γ-GT/ALT ratio in patients with HCC who were positive for HBV DNA

**Figure 4:**
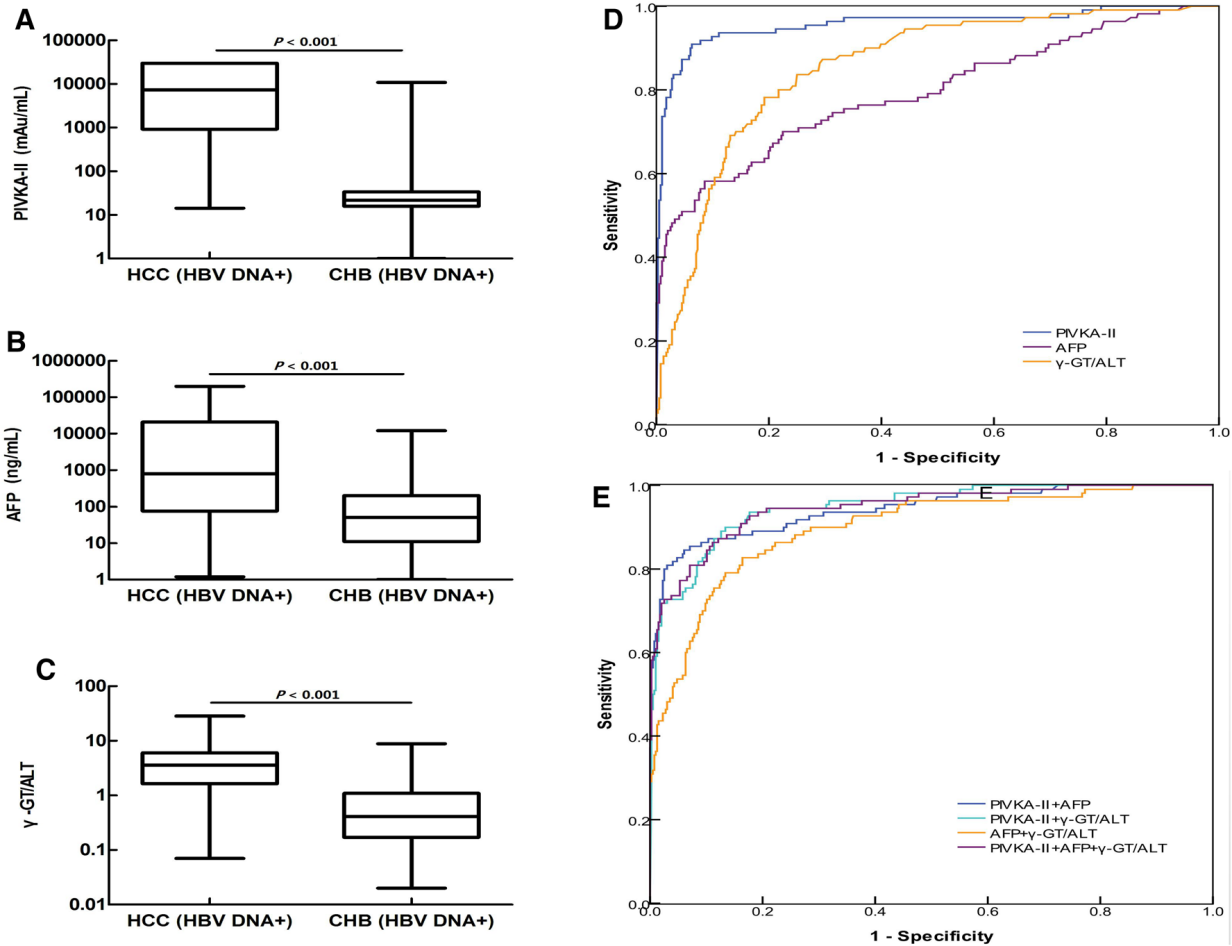
The diagnostic value of PIVKA-II, AFP, and the γ-GT/ALT ratio in patients with HCC and HBV DNA+ status. (**A**–**C**) PIVKA-II, AFP, and the γ-GT/ALT ratio were compared between patients with HCC and patients with CHB patients who were positive for HBV DNA. (**D**) ROC curves of PIVKA-II, AFP, and the γ-GT/ALT ratio in patients with HCC and HBV DNA+. Patients with CHB served as controls. (**E**) ROC curve of the combinations of PIVKA-II, AFP, and the γ-GT/ALT ratio in patients with HCC who were positive for HBV DNA. Patients with CHB served as controls.

Serum levels of PIVKA-II and AFP and the serum γ-GT/ALT ratio in patients with HCC who were positive for HBV DNA were higher than those in patients with CHB who were positive for HBV DNA (*P* < 0.001, *P* < 0.001, *P* < 0.001, respectively; Fig. 4A–C).

When the cut-off values of the γ-GT/ALT ratio and AFP and PIVKA-II levels were set as 1.580, 499.80 ng/mL, and 157.39 mAu/mL, respectively, in patients with HCC who were positive for HBV DNA, there were no significant differences in AUROCs between the γ-GT/ALT ratio and AFP (*P* = 0.069). However, the AUROCs of both AFP and γ-GT/ALT were lower than that of PIVKA-II (*P* < 0.001, *P* < 0.001, respectively; Fig. 4D).

In the diagnosis of HBV DNA+/HCC, when PIVKA-II was combined with AFP, the AUROC of PIVKA-II decreased (0.942 versus 0.957, respectively); however, there were no significant differences in AUROCs between combined diagnosis and single diagnosis (*P* = 0.432). For the diagnosis of HCC patients who were positive for HBV DNA, the AUROC of AFP combined with the γ-GT/ALT ratio was 0.896, which was higher than that of AFP alone (*P* = 0.002). The highest AUROC for the combined diagnosis of HCC with HBV DNA+ was 0.946; however, there was no significant difference in AUROC between this and PIVKA-II (*P* = 0.550; Fig. 4E).

### Diagnostic value of PIVKA-II, AFP, and the γ-GT/ALT ratio in patients with HBV DNA−/HCC

**Figure 5:**
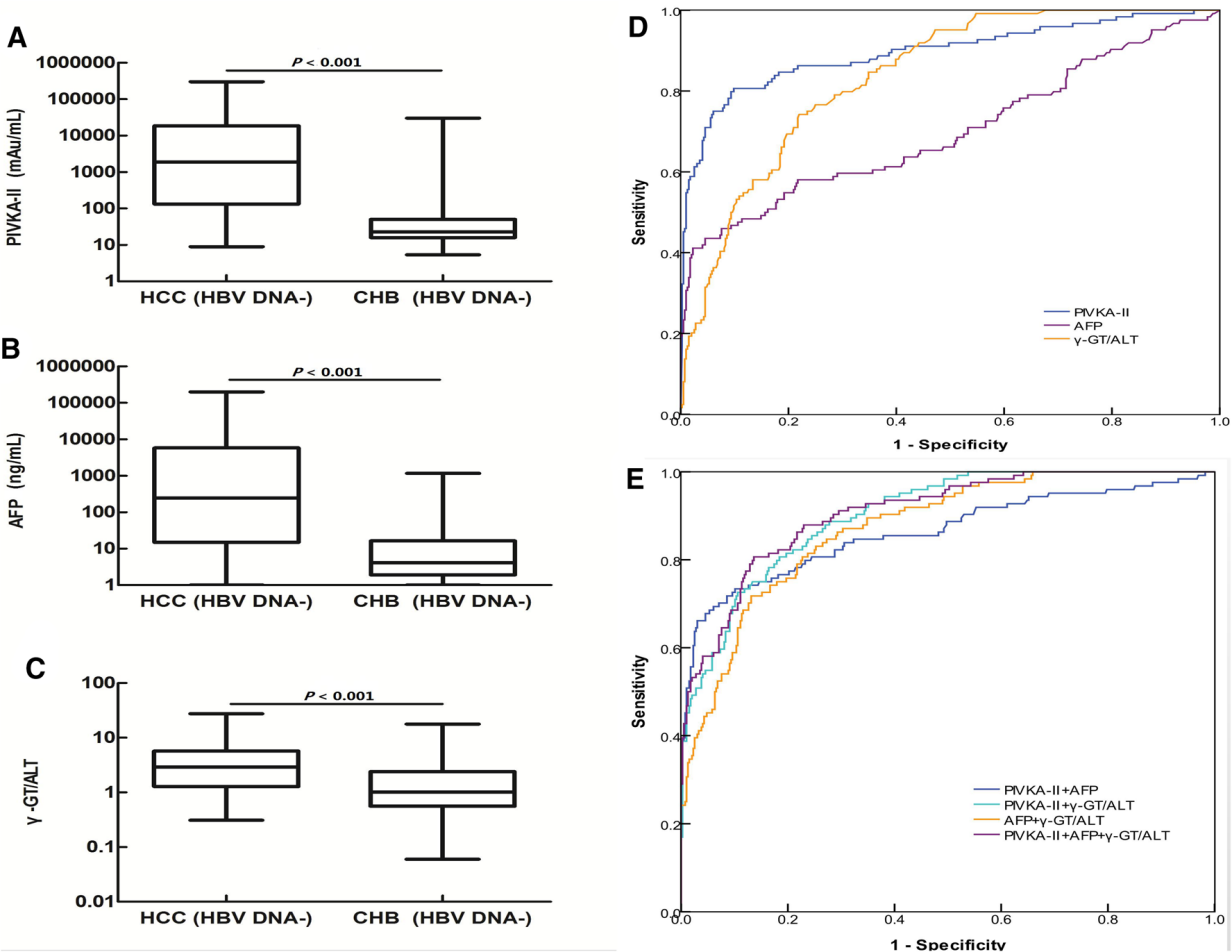
The diagnostic values of PIVKA-II, AFP, and the γ-GT/ALT ratio in patients with HCC who were negative for HBV DNA. (**A**–**C**) PIVKA-II, AFP, and the γ-GT/ALT ratio were compared between patients with HCC and patients with CHB who were negative for HBV DNA. (**D**) ROC curves of PIVKA-II, AFP, and the γ-GT/ALT ratio in patients with HCC who were negative for HBV DNA; patients with CHB served as controls. (**E**) ROC curve of the combinations of PIVKA-II, AFP, and the γ-GT/ALT ratio in patients with HCC who were negative for HBV DNA; patients with CHB served as controls.

Serum levels of PIVKA-II and AFP and the serum γ-GT/ALT ratio in patients with HCC who were negative for HBV DNA were higher than those in patients with CHB who were negative for HBV DNA (*P* < 0.001, *P* < 0.001, *P* < 0.001; Fig. 5A–C).

When the cut-off values of the γ-GT/ALT ratio, AFP levels, and PIVKA-II levels were set as 1.400, 761.45 ng/mL, and 87.63 mAu/mL, respectively, for patients with HCC who were negative for HBV DNA, the AUROCs of both the γ-GT/ALT ratio and PIVKA-II were higher than that of AFP (*P* < 0.001, *P* < 0.001, respectively). There was a significant difference between the AUROC of the γ-GT/ALT ratio and that of PIVKA-II (*P* = 0.027; Fig. 5D). Compared with patients with HCC who were positive for HBV DNA, the diagnostic value of PIVKA-II and AFP decreased in patients with HCC who were negative for HBV DNA (*P* = 0.007, *P* = 0.020). However, there were no significant differences in the diagnostic value of the γ-GT/ALT ratio between patients with HCC who were positive for HBV DNA and patients with HCC who were negative for HBV DNA (*P* = 0.492; Fig. 5D).

In patients with HCC who were negative for HBV DNA, when PIVKA-II combined with AFP was used to diagnose HBVDNA−/HCC, the AUROC of PIVKA-II decreased (0.863 and 0.895, respectively). However, when PIVKA-II combined withγ-GT/ALT ratio was used to diagnose HBVDNA−/HCC, the AUROC of PIVKA-II increased (0.902 and 0.895, respectively).There were no significant differences in AUROCs between combined diagnosis and single diagnosis (*P* = 0.283, *P* = 0.767, respectively). And the AUROC of AFP combined with the γ-GT/ALT ratio in the diagnosis of patients with HCC without HBV DNA was 0.873, which was higher than that of AFP alone (*P* < 0.001). For the combined diagnosis of HCC with HBV DNA−, the highest AUROC was 0.907, However, there was no significant difference in AUROC between this and PIVKA-II (*P* = 0.620; Fig. 5E). The diagnostic performance of PIVKA-II, AFP, and the ratio of γ-GT/ALT for HCC with and without HBV DNA positivity are shown in Table 5.

**Table 5:** Performance values of PIVKA-II, AFP, and the γ-GT/ALT ratio in patients with HCC with different HBV DNA statuses.

	HCC (HBV DNA+)						HCC (HBV DNA−)					
	Cut-off	AUROC (95% CI)	SEN (%)	SPE (%)	PPV (%)	NPV (%)	Cut-off	AUROC (95% CI)	SEN (%)	SPE (%)	PPV (%)	NPV (%)
PIVKA-II (mAu/mL)	157.39	0.957 (0.932–0.983)	90.90	93.70	80.00	97.38	87.63	0.895 (0.857–0.933)	80.60	90.20	71.94	93.70
AFP (ng/mL)	499.80	0.795 (0.741–0.848)	58.20	91.40	65.31	88.73	761.45	0.699 (0.638–0.759)	43.50	95.50	75.00	84.38
γ-GT/ALT	1.58	0.855 (0.817–0.893)	78.20	80.80	53.09	90.40	1.40	0.837 (0.801–0.872)	74.20	78.00	51.40	90.62
PIVKA-II & AFP	NA	0.942 (0.915–0.969)	84.50	93.90	79.49	95.63	NA	0.863 (0.819–0.907)	73.40	89.90	69.47	91.52
PIVKA-II & γ-GT/ALT	NA	0.948 (0.926–0.970)	90.00	86.60	65.13	96.89	NA	0.902 (0.874–0.931)	80.60	81.60	57.80	93.08
AFP & γ-GT/ALT	NA	0.896 (0.862–0.931)	82.70	83.60	58.33	94.57	NA	0.873 (0.839–0.906)	71.80	86.90	63.12	90.77
PIVKA-II & AFP & γ-GT/ALT	NA	0.946 (0.921–0.970)	86.40	88.90	68.35	95.91	NA	0.907 (0.878–0.936)	80.60	86.40	64.94	93.44

*HCC* hepatocellular carcinoma, *SEN* sensitivity, *SPE* specificity, *PPV* positive predictive value, *NPV* negative predictive value, *PIVKA-II* protein induced by vitamin K absence or antagonist II, *AFP* alpha-fetoprotein, *γ-GT/ALT* ratio of γ-glutamyl transferase to alanine transaminase, *NA* not applicable.

## Discussion

AFP is the most commonly applied tumour marker in HCC; however, its sensitivity varies (33–85%, mean: 56.3%), which is not optimal for early diagnosis of HCC^38^. Therefore, there is an urgent need to identify better serological biomarkers, either alone or in combination with AFP, for the early diagnosis of HCC. In particular, markers with better ability to discriminate HCC from hepatitis viral infection are required. PIVKA-II is known to be abnormally expressed in liver tissues and elevated in the serum of patients with HCC. The sensitivity and specificity of PIVKA-II for the diagnosis of HCC may be superior to AFP, but the roles of PIVKA-II in HBV-related HCC might vary a lot considering different mechanisms of hepatocarcinogenesis among different aetiologies^39^. PIVKA-II coupled with AFP has been routinely used for HCC screening. However, these indicators, along with false positive or false negative sometimes^40^. New markers and more cost-effective, appropriate combination of these markers for the diagnosis and surveillance for HBV-related HCC are urgently needed.

γ-GT is generally considered a biomarker of alcoholic hepatitis^41,42^. In HCC, particularly advanced HCC, cancer cells produce a certain amount of γ-GT, which is abnormally elevated in the serum of patients with HCC^42^, however, γ-GT is also increased in patients with CHB. Therefore, HCC and CHB cannot be distinguished by measuring serum γ-GT levels alone. ALT, which does not increase or even decreases in HCC, is more sensitive and is increased to varying degrees in almost all types of hepatitis. Therefore, it is necessary to evaluate whether the γ-GT/ALT ratio can be used as a diagnostic marker of HBV-related HCC.

In this study, we found that PIVKA-II and AFP levels and the γ-GT/ALT ratio were significantly higher in patients with HBV-related HCC than in patients with CHB. Moreover, the diagnostic values of PIVKA-II and the γ-GT/ALT ratio were significantly higher than that of AFP, and the γ-GT/ALT ratio and PIVKA-II had the same value in the diagnosis of early-stage HCC. Therefore, the ratio of γ-GT/ALT and the levels of PIVKA-II in the serum could be used for the diagnosis of early-stage HCC. Further analysis showed that the diagnostic sensitivity of PIVKA-II was significantly higher than that of the γ-GT/ALT ratio and AFP for HCC, HCC (HBV DNA+), and HCC (HBV DNA−), whereas the γ-GT/ALT ratio and the level of PIVKA-II in the serum had the same sensitivity for the diagnosis of early-stage HCC. Moreover, the diagnostic sensitivity of the γ-GT/ALT ratio was higher than that of AFP in early-stage HCC, HCC, HCC (HBV DNA+), and HCC (HBV DNA−). Therefore, the ratio of γ-GT/ALT was a useful biomarker for the diagnosis of HBV-related HCC. In addition, the cost for detection of γ-GT and ALT in the laboratory is very low, making these markers cost-effective indicators for the clinical diagnosis of HBV-related HCC.

Serum levels of PIVKA-II and AFP in patients with HCC who were positive for HBV DNA were significantly higher than those in patients with HCC who were negative for HBV DNA, indicating that continuous replication of HBV may promote the formation of PIVKA-II and AFP during the malignant transformation of hepatocytes. These changes may promote the occurrence of HCC in patients with CHB^16^. For patients with HCC without detectable HBV DNA, elevation of PIVKA-II and AFP was relatively lower than that in patients with HCC who were positive for HBV DNA. Thus, the diagnostic values of PIVKA-II and AFP were significantly lower in patients who were negative for HBV DNA than in patients who were positive for HBV DNA. In contrast, there were no significant differences in the diagnostic value of the γ-GT/ALT ratio among these patients, suggesting that the γ-GT/ALT ratio may be more useful in the diagnosis of patients with HCC who were negative for HBV DNA.

When the γ-GT/ALT ratio, AFP, and PIVKA-II were used in combination for the diagnosis of early-stage HCC, HCC, HCC with HBV DNA+, and HCC with HBV DNA−, the diagnostic performance of the γ-GT/ALT ratio combined with AFP or PIVKA-II was better than that of the biomarkers alone. The AUROCs of PIVKA-II combined with γ-GT/ALT ratio for the diagnosis of early-stage HCC, HCC, HCC with HBV DNA+, and HCC with HBV DNA− were higher than those of PIVKA-II combined with AFP and AFP combined with the γ-GT/ALT ratio, implying improvement of the diagnostic value. Therefore, the combination of the γ-GT/ALT ratio and PIVKA-II may be useful for the differential diagnosis of HBV-related HCC and other benign liver diseases.

Our results also showed that the γ-GT/ALT ratio was positively correlated with tumour size, indicating that the γ-GT/ALT ratio may be another indicator for monitoring the progression of HBV-related HCC, similar to PIVKA-II and AFP. Therefore, further studies are necessary to explore the application of the γ-GT/ALT ratio in greater detail and assess the use of this marker in the monitoring of patients with HCC.

In conclusion, our study found the γ-GT/ALT ratio might be a potential biomarker for screening HBV-related HCC patients and patients with CHB and in combination with the AFP and PIVKA-II could improve the diagnostic value. Thus, our findings may add an important non-invasive marker for diagnosis of HBV-related HCC. Our study results also showed a positive correlation of tumour size with γ-GT/ALT ratio, indicating that it may be a useful index in monitoring patients for progression of HBV-related HCC. However, this study did not make a more detailed staging of advanced HCC, which needs to be further demonstrated by follow-up experiments. In addition, the current study had some limitations, such as retrospective research, single-centre nature and small sample. These factors will lead to statistical errors regarding sensitivity, specificity and accuracy.

## Data Availability

The datasets generated during and analyzed during the current study are available from the corresponding author on reasonable request.
